# Characterization of the complete chloroplast genome sequence of Chinese endemic species of *Nouelia insignis* (Hyalideae, Asteraceae) and its phylogenetic implications

**DOI:** 10.1080/23802359.2021.1921629

**Published:** 2022-04-01

**Authors:** Xiaofeng Liu, Maoyun Han, Jia Chen, Xun Zhang, Zhiyu Chen, Yi Tang, Tianmeng Qu, Chunping Huang, Shuhua Yu, Zhixi Fu

**Affiliations:** a College of Life Sciences, Sichuan Normal University, Chengdu, China; b Chengdu Foreign Languages School, Chengdu, China; c Jinjiang Experimental School, Chengdu, China; d Institute of Application and Development of Plant Resources, Sichuan Normal University, Chengdu, China; e Sustainable Development Research Center of Resources and Environment of Western Sichuan, Sichuan Normal University, Chengdu, China; f Natural Resources Bureau of Maoxian, Maoxian, China

**Keywords:** *Nouelia insignis*, complete chloroplast genome, phylogenomics analysis

## Abstract

This study was the first report complete chloroplast genome of *Nouelia insignis* (Asteraceae, Hyalideae), the large shrubs to small trees endemic to China. The circular whole cp genome of *N. insignis* was 151,524 bp in length, containing a large single-copy (LSC) region of 83,145 bp and a small single-copy (SSC) region of 18,261 bp. These two regions were separated by a pair of inverted repeat regions (IRa and IRb), each of them 25,060 bp in length. A total of 135 functional genes were encoded, consisting of 89 protein-coding genes, 38 tRNA genes, and eight rRNA genes. The overall GC content of the chloroplast genome sequence was 37.8%, and the GC contents of the LSC, SSC, and IR regions were 35.9, 31.5, and 43.2%, respectively. The phylogenetic analysis by the Bayesian analysis showed that the species of *N.* *insignis* was sister group with *Gerbera jamesonii* by strong support values, and thus was closely related to members of subfamilies of Cichorioideae and Pertyoideae. These results will be useful for the future studies of Asteraceae in the worldwide.

The species of *Nouelia insignis* Franch. (Asteraceae, Hyalideae) is a genus endemic to southwest China (Hind 2007; Gao and Hind 2011; Gong et al. 2011; Fu et al. 2016). They are narrowly and allopatrically distributed species, separated by the important biogeographic boundary Tanaka Line in Southwest China (Zhao and Gong 2015). Genetic knowledge of *N. insignis* would provide information for protection of this wild germplasm resource. Here, we obtained the complete plastome of *N. insignis* by Illumina sequencing technology (San Diego, CA). The complete plastome reported here will contribute to the further studies on the phylogenetic analysis of *N. insignis*.

Fresh leaves of *N. insignis* were collected from Wulaxi village (101°39′36″E, 28°37′12″N), Jiulong county, Sichuan Province, China. A specimen was deposited at the botany herbarium of Sichuan Normal University, SCNU (Associate Professor, Dr. Zhixi Fu, fuzx2017@sicnu.edu.cn) under the voucher number Z.X. Fu 2862. High quality total genomic DNA was extracted from ca. 6 cm^2^ sections of the silica-dried leaf using improved Tiangen Plant Genomic DNA Kits, add the 4 μl RNAseA and 20 μl Proteinase K after incubated (65 °C). Total DNA was directly constructed short-insert of 150 bp in length libraries and sequenced on the Illumina Genome Analyzer (Hiseq 2000) based on the manufacturer’s protocol (Illumina, San Diego, CA) by ORI-GENE (Beijing, China). Generally, more than 5.2 Gb of data was obtained for complete cp genome of *N. insignis*; *De novo* assembly of CLC Genomic Workbench v11 (CLC Bio, Aarhus, Denmark) and consensus sequence of Geneious R11.1.5 (Biomatters Ltd., Auckland, New Zealand) with referenced chloroplast genome sequence of *Gerbera jamesonii* (accession no.: MN087227). The chloroplast genome was annotated using a web-based annotation program GeSeq (https://chlorobox.mpimp-golm.mpg.de/geseq.html) and editing by manual and imagining with OGDraw v1.2 (Lohse et al. 2013).

The complete chloroplast genome of *N. insignis* was 151,524 bp in length and a typical circular structure. The genome sequence data that support the findings of this study are openly available in GenBank of NCBI at https://www.ncbi.nlm.nih.gov under the accession no. MT386594. The associated BioProject, SRA, and Bio-Sample numbers are PRJNA694507, SRP303716, and SAMN17526016 (SRS8144786), respectively. It includes a pair of inverted repeat (IR) of 25,060 bp divided by a large single-copy (LSC) region of 83,145 bp and a small single-copy (SSC) region of 18,261 bp. The general G + C content was 37.8% in the whole sequence and the corresponding values in the LSC, SSC, and IR regions are 35.9%, 31.5%, and 43.2%, respectively. The whole genome contained 135 genes, including 89 protein-coding genes, eight ribosomal RNA genes, and 38 tRNA genes, nevertheless, 114 unique genes, 20 genes duplicated in the IRs. In addition, among the annotated chloroplast genomic sequence, 15 genes possessed only single intron, two genes (*ycf3* and *clpP*) possessed two introns.

To identify the phylogenetic position of *N. insignis*, we used a total of 30 additional complete cp genomes of the family Asteraceae and one outgroup taxa to clarify the phylogenetic position of *N. insignis* (Figure 1). All of the cp genome sequences were aligned in MAFFT (Katoh and Standley 2013). A maximum-likelihood analysis based on the GTRGAMMA model was performed with Bayesian method on the CIPRES (Miller et al. 2010; Ronquist et al. 2012) using 1000 bootstrap replicates. The phylogenetic analysis of the cp genome dataset recovers the similar clades as in previous phylogenetic work (Panero and Funk 2008; Panero et al. 2014; Fu et al. 2016). The Bayesian inference (BI) result with 100% bootstrap showed that *N. insignis* has a close sister relationship with the genus *Gerbrea* (Figure 1). The complete cp genome sequence of *N. insignis* will be the valuable resource for future studies on taxonomy and phylogeny of family Asteraceae and provides useful molecular data for further phylogenetic and evolutionary analysis.

**Figure 1.**
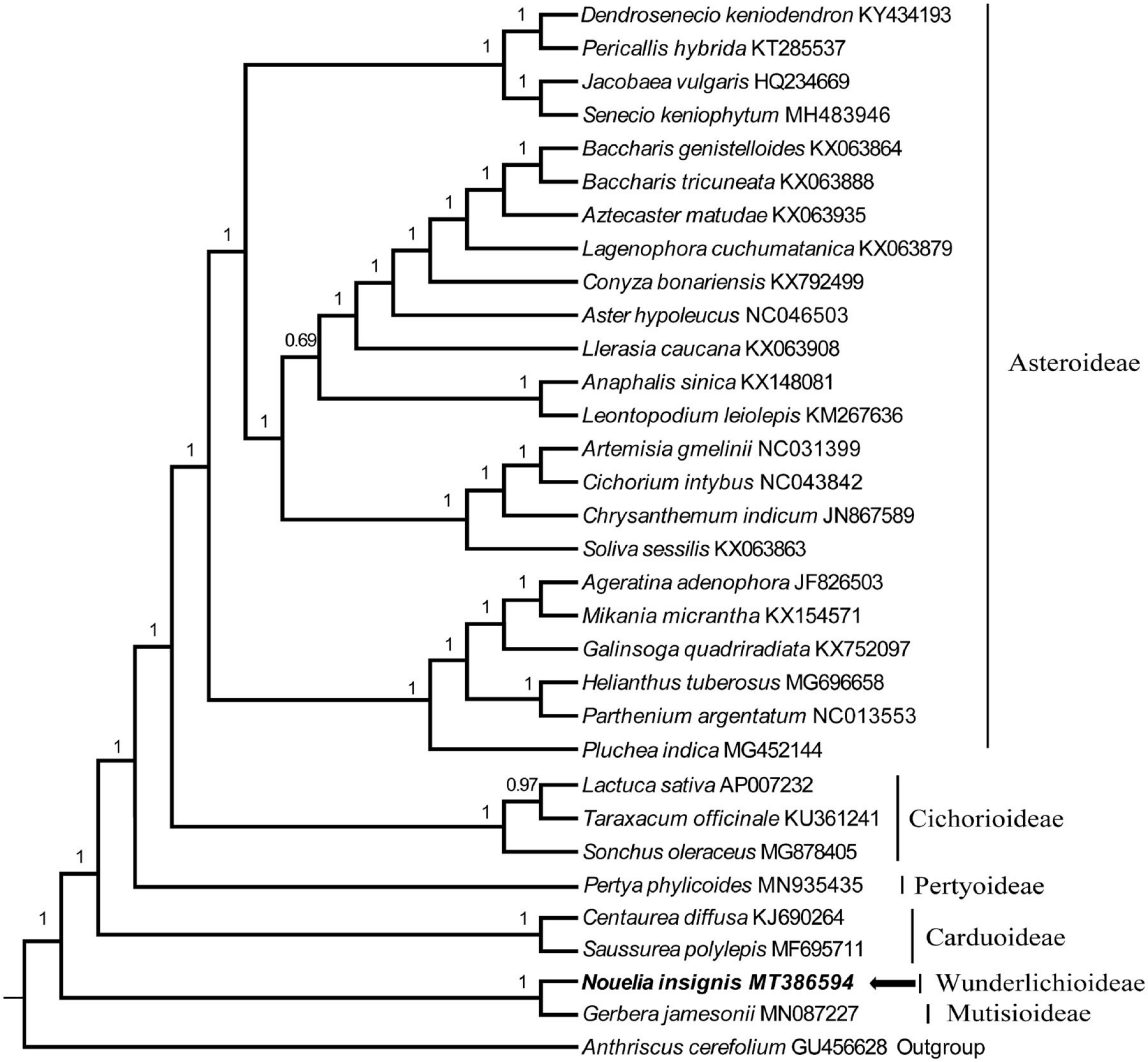
The Bayesian inference (BI) phylogram inferred from 31 chloroplast genomes in Asteraceae (bootstrap value are indicated on the branches). The position of *Nouelia insigni*s is in bold.

## Data Availability

The data that newly obtained at this study are openly available in the NCBI (https://www.ncbi.nlm.nih.gov/) under accession number of MT386594. Raw sequencing reads were deposited in SRA with BioProject accession (PRJNA694507) (https://www.ncbi.nlm.nih.gov/bioproject/PRJNA694507).
